# Social and emotional development of disadvantaged students and its relationship with academic performance: evidence from China

**DOI:** 10.3389/fpsyg.2023.1170656

**Published:** 2023-05-09

**Authors:** Chunhan Huang, Xiaodong Zeng

**Affiliations:** Faculty of Education, Beijing Normal University, Beijing, China

**Keywords:** academic performance, social and emotional skills, self-efficacy, teacher-student relationship, structural equation modeling

## Abstract

This research examined the relationships between students’ academic performance and their social and emotional skills in China, as well as the mediation pathways from the perspective of connectedness and social cognitive theory. A sample of 5,703 fourth to sixth graders from less affluent areas was investigated in a large-scale survey. The results indicated that students’ academic performance had salient positive connections with their socio-emotional skills. Structural equation modeling results revealed that both students’ perception of themselves (self-efficacy) and their relation with key persons (teacher-student relationship) played mediated roles in the association between academic achievement and social and emotional skills with the indirect path accounting for 66.66% of the total effect. This study highlights the important role of socioemotional skills in academic performance and suggests the need for further research to develop effective strategies and interventions for socioemotional development in Chinese students.

## 1. Introduction

The relationship between students’ cognitive and social and emotional development has been the subject of intensive research in recent decades. Empirical evidence has shown that social and emotional skill is an individual characteristic that is critical for counteracting the negative effects of exposure to adversity or risk (Domitrovich et al., 2017). Children who grow up in a disadvantaged background, relative to their more affluent peers, are at heightened risk for exhibiting poorer social and emotional skills (McCoy et al., 2018; Sibley et al., 2019). And they are disproportionately at risk of poor academic performance (Murry et al., 2011). Most studies have focused on how social–emotional skills affect academic attainment, however, far less is known about the reciprocal effect of academic performance on socioemotional skills.

Our analysis mainly builds on two strands of research. First, prior empirical evidence suggested that social–emotional skills and academic performance are not separable, as they mutually influence each other. One type of skill can help foster the other skills over time (Cunha and Heckman, 2007; Cunha et al., 2010). A second strand of research considers the influence mechanism among attitude, skill and behavior (Sitzmann and Yeo, 2013).

## 2. Literature review

### 2.1. Social–emotional skills and academic performance

Social–emotional skills are commonly defined as the knowledge, attitude and skills to understand and manage emotions, set and achieve positive goals, feel and show empathy for others, establish and maintain positive relationships, and make responsible decisions (Weissberg et al., 2015). These skills can be framed into two domains: intrapersonal skills that focus on effective functioning as an individual, and interpersonal skills that relate to successfully build and maintain positive interactions with others (Collaborative for Academic, Social, and Emotional Learning [CASEL], 2016).

Numerous factors affect social and emotional skills. Learning contexts including families (Cabrera et al., 2007; Kiernan and Huerta, 2008; Cunha et al., 2010; Baxter and Smart, 2011), schools (Covay and Carbonaro, 2010), and communities (Sampson et al., 1999; Lewis, 2006) play critical roles in molding social and emotional development. Social–emotional abilities and academic achievement are inextricably linked, since they interact and impact one another.

### 2.2. Student-teacher relationship

Human attitudes, skills, and behaviors are often shaped by both internal personal factors and external environmental factors (Bandura, 1997, 2002; Honicke and Broadbent, 2016; Schunk and DiBenedetto, 2020). As Baker (1999) and Elias (1987) articulated, if we regard education to be a socially situated process, any attempt to explain this phenomenon should consider elements at the individual as well as the environmental levels.

Teachers and teacher-student relationships constitute an important part of the internal factors for elementary school students. Attachment theory indicates that social connectedness is essential for children to internalize social standards and to develop recognition for social institutions (Deci and Ryan, 1985; Baker, 1999). From the standpoint of socialization, the nature of teacher-student interaction is operating as socializing agents. Teachers play a role in socialization by addressing students’ need to belong (Connell and Wellborn, 1991; Wentzel, 1998); and regulating emotional, behavioral, and academic skills (Birch and Ladd, 1998; Pianta, 1999; Yowell and Smylie, 1999). Muller et al. (1999) found that teachers weigh a variety of factors, including expectations about students’ ability level, classroom conduct, and peer group, when deciding to invest in a relationship with a student, leading to reciprocal effects between teacher–student relationships and students’ abilities and performance.

Relationships with teachers can either facilitate or impede students’ learning and other social behavior, while students’ abilities and performance can either enhance or degrade the quality of relationships with their teachers. Dubow and Tisak (1989) found that students who reported more support from their teachers had fewer problem behaviors. And those that are characterized by a high level of warmth and trust may change students’ behaviors favorably (Hamre and Pianta, 2001; Wubbels and Brekelmans, 2005; Baker et al., 2008; Heatly and Votruba-Drzal, 2017; Ansari et al., 2020).

### 2.3. Self-efficacy

Among the internal personal factors, self-efficacy is considered to influence all aspects of a person’s life. It refers to an individual’s perception that they possesses the necessary skills to complete a designated task or achieve a goal (Bandura, 1986). The idea of self-efficacy is founded on the assumption that people’s performance in various life situations is influenced not only by their capacities but also by their belief in the strength of those capacities (Bandura, 1997). People’s beliefs in their capabilities can predict their performance better than their actual level of capability since these beliefs influence how and to what degree they utilize their knowledge and skills (Bandura, 1977; Chernyshenko et al., 2018).

### 2.4. The present study

**Figure 1:**
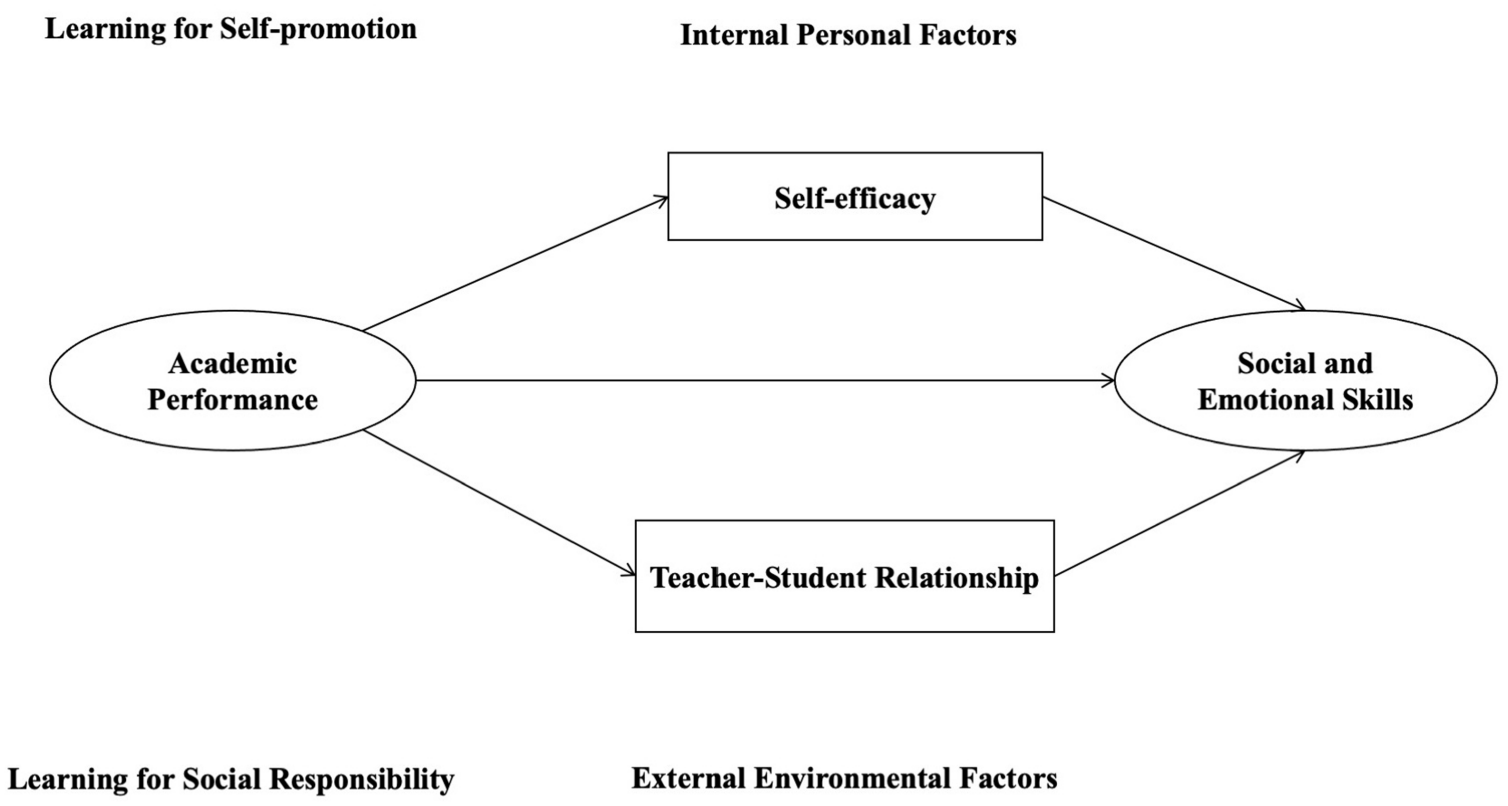
Conceptual framework of the present study.

In this study, we examined the relationship between academic attainment and social–emotional skills using a conceptual framework (see Figure 1) based on connectedness and social cognitive theory. We categorized social–emotional skills into intrapersonal and interpersonal skills and identified two pathways: learning to for self-improvement and learning for social responsibility. The connectedness perspective suggested that students have needs to belong, in school settings, connections with teachers might shape students’ performance. According to the social cognitive theory, self-efficacy played as a strong predicter for performance, as students’ belief about themselves can influence their school performance. Our analysis leveraged a uniquely detailed dataset that links students’ self-reported survey measures of social–emotional skills to administrative data of standardized test scores. The context for this analysis is in underdeveloped regions, from fourth to sixth grade, the final 3 years of primary education in China. The period of grade four to grade six is noteworthy because developmental and contextual changes occurring in this phase may lay the foundations for a successful early adolescent transition.

## 3. Materials and methods

### 3.1. Participants and procedures

This study was a cross-sectional design and used data collected from an investigation in China in 2019. Students in fourth through sixth grade from 89 schools were selected from 3 underdeveloped counties of Gansu province in northwest China and Heilongjiang province in northeast China. All of the three counties were on the “national poverty county list” and located in one of the 14 “poverty blocks,” mostly located in ethnic minority areas, western China, or remote and mountainous areas. Participants’ demographic information across the sample is reported in Table 1. All primary schools in these 3 counties were included in this study. All of the fourth to sixth grade students from each school were invited to participate in this survey and the total sample size was 5,703 students. Self-completion questionnaires were used to obtain data on social and emotional abilities, while standardized exam results at the county level were used to collect data on academic performance. Self-reported assessments of social emotional skills and standardized academic tests were conducted at the end of 2018–2019 academic year.

**Table 1:** Sample demographic characteristics.

	Sex		Grade		
	Girls	Boys	Fourth	Fifth	Sixth
*N*	2,478	2,782	2,364	2,138	1,072
Percent	47.11%	52.89%	42.42%	38.36%	19.23%

Informed consent was obtained from all students, their teachers, and their parents or custodians.

### 3.2. Measures

#### 3.2.1. Social and emotional skills

Social and emotional skills were measured using the subscales of “Social Adaptability Questionnaire” developed by National Children’s Study of China project (Dong and Lin, 2011). Dong and Lin’s prior studies have established the instrument with high internal consistency reliability, retest reliability, structure validity, clinical discrimination validity, and criterion-related validity. Their team organized 4 nationwide pretests with more than 20,000 participants and conducted validity and reliability studies based on normal and clinical samples.

In the present study, four dimensions were included to evaluate social and emotional skills, they were self-control, sense of achievement, learning attitude, and prosocial and aggressive behavior. And each dimension was assessed through widely used 4-point or 5-point scales. Higher composite values suggested better personality traits of students. The subscales for assessing self-control, sense of achievement, and prosocial and aggressive behaviors were 4-point Likert scales (1 = definitely does not apply, 4 = definitely applies). One example of the items is “I believe that I can successfully complete the tasks assigned by my teacher.” Learning attitude was a 5-point Likert scale, from 1 strongly disagree to 5 strongly agree. One example of the items is “I study very hard.” Items were alternately expressed as a positive or a negative statement to minimize response biases. The reliability (Cronbach’s alpha) of the social and emotional skills measures in this study was 0.777. The confirmatory factor analysis (CFA) model fit indices were indicating typically good construct validity: χ^2^ (2) = 19.484, *p* < 0.001; CFI = 0.997; TLI = 0.990; RMSEA = 0.041; SRMR = 0.012; and factor loadings were greater than 0.5 (see Table 2).

**Table 2:** Reliability and validity analysis of the study measures.

Measure	Reliability	Construct validity						
	Cronbach’s alpha	Range of factor loadings	χ^2^	DF	CFI	TLI	RMSEA	SRMR
Social–emotional skills	0.777	0.569–0.722	19.484***	2	0.997	0.990	0.041	0.012
-Self-control	0.733	0.520–0.608	61.756***	5	0.988	0.977	0.046	0.018
-Sense of achievement	0.584	0.472–0.545	Saturated model					
-Learning attitude	0.740	0.521–0.682	54.455***	5	0.991	0.981	0.043	0.016
-Prosocial behavior	0.904	0.575–0.751	936.616***	54	0.966	0.958	0.056	0.029
Teacher-student relation	0.764	0.421–0.674	73.958***	9	0.991	0.985	0.037	0.016
Self-efficacy	0.798	0.520–0.683	29.554***	9	0.998	0.996	0.021	0.010
Academic performance	0.835	0.755–0.771	Saturated model					

***p < 0.001; fit indices of the saturated model could not be examined, only factor loadings were reported.

#### 3.2.2. Academic performance

We used the county level test scores in Chinese literature, mathematics, English, and science to evaluate academic performance. At the end of each semester in the elementary phase of education in China, students take standardized assessment tests in core curriculum areas as a means to assess their learning status. Raw scores were converted to standardize the scores in school level in the current study, considering that test papers from different schools would be marked by different raters, even the content of tests was the same within each county. We built a latent variable “academic performance” based on the scores in four core curriculum areas, with an internal consistency reliability at 0.856. The model for academic performance was also saturated, and the factor loadings of Chinese literature, English, and mathematics were 0.758, 0.755, and 0.771, respectively, which were found to be high (see Table 2).

#### 3.2.3. Teacher-student relationship

Teacher-student relationship scale captured students’ perception of the quality of the relationship with his/her present homeroom teacher. The scale consisted of six self-report Likert scale items ranging from 1 (strongly disagree) to 5 (strongly agree), with higher total scores indicating more positive perceptions of the teacher-student relationship. One example item was “the homeroom teacher is very approachable and students like the homeroom teacher.” The internal consistency reliability of the teacher-student relationship scale as estimated by Cronbach’s alpha was acceptable at 0.764, the construct validity assessed by CFA model was considered high (χ^2^ (9) = 73.958, *p* < 0.001; CFI = 0.991; TLI = 0.958; RMSEA = 0.037; SRMR = 0.016; with factor loadings larger than 0.4). The final scores adopted in the analysis were transferred into the same scale ranging from 1 to 4 (see Table 2).

#### 3.2.4. Self-efficacy

Self-efficacy scale measured the degree of student perceived ability when finishing a task or facing a challenge. This scale consisted of six self-report items, such as “when encountering problems in daily life, I often say ‘I can make it’”, “when faced with different difficult and easy tasks, I believe that I can complete the difficult one,” etc. Students rated on a 4-point Likert scale from 1 (definitely does not apply) to 4 (definitely applies). Cronbach’s alpha for internal consistency reliability of the self-efficacy scale was at 0.798. The CFA model fit indices indicated that this measure was of high construct validity: χ^2^ (9) = 29.554, *p* < 0.001; CFI = 0.998; TLI = 0.996; RMSEA = 0.021; SRMR = 0.010. The factor loadings range was from 0.520 to 0.683 (see Table 2).

Moreover, in this survey, demographic information including students’ age, gender, socioeconomic status, and other background data, such as whether on board or not, were captured. SES contained parental education levels, parental occupations, family possession, family parenting activities.

### 3.3. Statistical analyses

We first conducted descriptive statistics analysis and Pearson’s correlation analysis to examine the distributions of these study variables. Then we examined the hypothesized mediational model with structural equation modeling (SEM) under robust maximum likelihood estimation (ML). Considering the potential influence of students’ grade, school and socioeconomic status, we controlled for these factors which had a significant effect on the model.

To evaluate the fit of the models, multiple recommended indices were adopted in this study. For these indices, taking the sample size of this study into consideration, CFI (Comparative Fit Index) and TLI (Tucker-Lewis Index) values close to 0.95 were typically considered good, and RMSEA (Root Mean Square Error of Approximation) value close to 0.06 and SRMR (Standardized Root mean Squared Residual) value below 0.08 represented a relatively good model (Browne and Cudeck, 1993; Hu and Bentler, 1999). The chi-square statistic was reported in the results but was not used to evaluate the model fit due to its sensitivity to sample size (Panayiotou et al., 2019). At last, considering that mediational analyses based on cross-sectional design might be biased when estimating parameters, significance of the mediation effect was examined with the bootstrap resampling technique using 500 replications.

## 4. Results

### 4.1. Preliminary analysis

All descriptive statistics (see Table 3) were based on original data which contain missing values; while all other analyses were based on data without missing values (*n* = 4,809). Significant positive associations were found between all dimensions. In our analysis, although not shown, we found that among the factors of student’s grade, school and socioeconomic status, only student’s grade and school had a statistically significant influence on the model. To avoid the influence of school and grade fixed effects, we controlled them in the subsequent mediation effect analysis.

**Table 3:** Descriptive statistics.

Variables	*N*	Mean	SD	Minimum	Maximum
Social–emotional skills					
-Self-control	5,554	3.234	0.602	1.000	4.000
-Sense of achievement	5,452	3.245	0.637	1.000	4.000
-Learning attitude	5,571	3.466	0.508	1.000	4.000
-Prosocial behavior	5,599	3.455	0.467	1.511	4.000
Teacher-student relation	5,632	3.540	0.532	1.000	4.000
Self-efficacy	5,663	3.198	0.564	1.000	4.000
Academic performance					
-Chinese literature	5,672	0.000	0.983	−7.521	2.426
-Math	5,670	0.000	0.983	−4.934	2.850
-English	5,667	0.000	0.983	−6.015	2.733

### 4.2. Mediation effects analysis

To examine the mediation effects of self-efficacy and teacher-student relationship on the relation between academic achievement and social and emotional skills, our analysis proceeded in two steps. First, we ran a direct effect model to test the effect of academic performance on social and emotional skills, after controlling for sex, grade, and school (see Table 4). The resulting model fit indices were good:χ^2^ (31) = 328.169, *p* < 0.001; CFI = 0.973; TLI = 0.963; RMSEA = 0.0045; SRMR = 0.034. The results revealed that the higher the academic achievement, the better the students’ social and emotional competence (*β* = 0.178, *p* < 0.001).

**Table 4:** Direct effect of academic performance on social–emotional skills.

	Coefficient	*P*-value
Academic performance	0.178	0.000
Sex	−0.044	0.006
Grade	−0.077	0.000
School-effect	0.015	0.361

**Figure 2:**
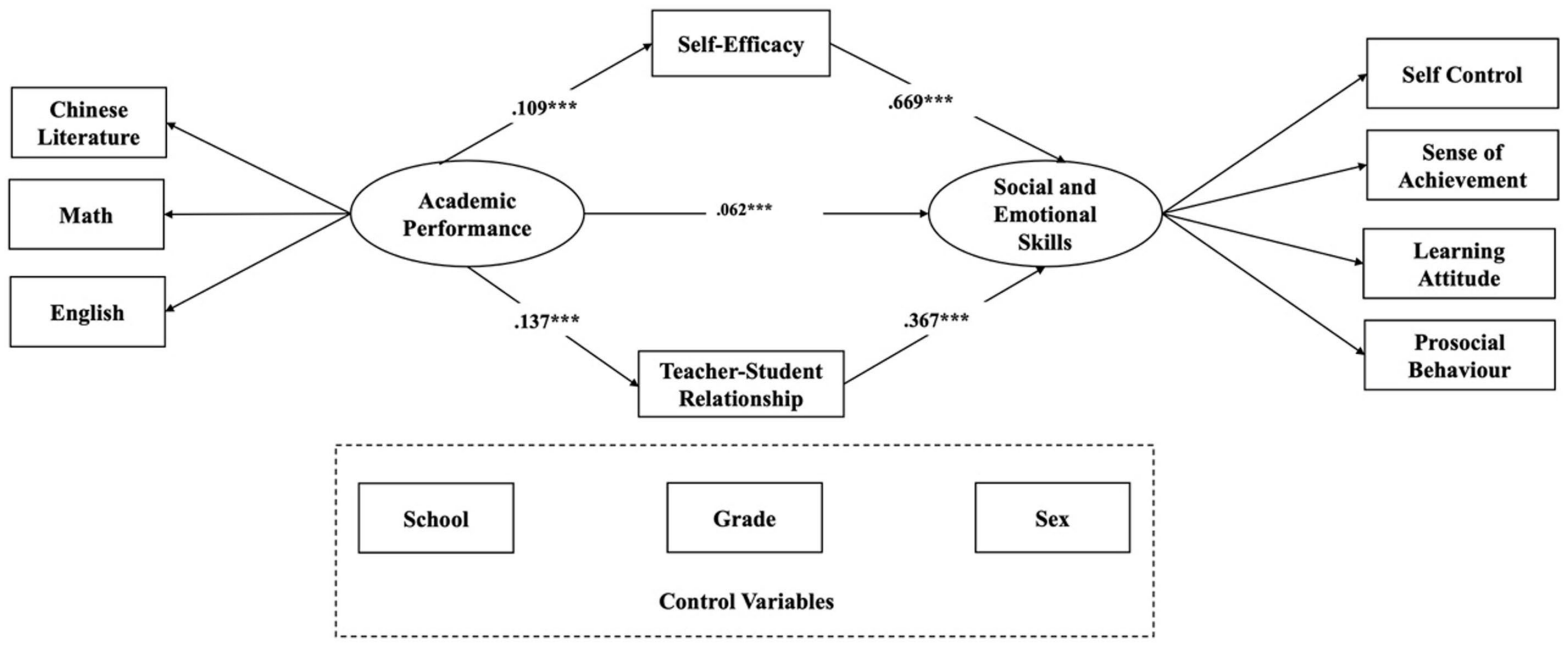
Results of mediation model. ***p < 0.001; Coefficients are standardized.

Second, we performed an indirect effect model analysis to test the mediational roles of self-efficacy and teacher-student relationship. The results showed statistically significant standardized path coefficients. Academic performance predicted self-efficacy (*β* = 0.109, *p* < 0.001), teacher-student relationship (*β* = 0.137, *p* < 0.001), and social and emotional skills significantly; self-efficacy and teacher-student relationship predicted social emotional skills significantly (*β* = 0.669, *p* < 0.001; *β* = 0.367, *p* < 0.001; respectively; see Figure 2). Indices showed that the mediational model fit was acceptable (χ^2^ (45) = 787.317, *p* < 0.001; CFI = 0.957; TLI = 0.939; RMSEA = 0.059; SRMR = 0.039).

At last, we examined the indirect effect with bootstrap resampling technique for robustness. All bias-corrected 95% confidence intervals did not include zero, indicating that the two indirect paths were still significant (see Table 5). 83.80% of the variance in social and emotional skills was predicted by the overall model. The results also indicated that this was a partial mediation effect model, and the mediating effect of the indirect path represents 66.66% of the total effect (see Table 6). The positive relationship between academic achievement and social and emotional skills was mediated by self-efficacy and teacher-student relationship.

**Table 5:** Robustness test of mediational effect based on bias-corrected bootstrap analysis.

Paths	Coefficient	Bootstrap SE	Bias-corrected 95% CI	
			Lower	Upper
AcdP-- > SelE-- > SocE				
AcdP-- > SelE	0.079	0.014	0.057	0.106
SelE-- > SocE	0.444	0.014	0.417	0.473
AcdP-- > TeaS-- > SocE				
AcdP-- > TeaS	0.124	0.013	0.095	0.150
TeaS-- > SocE	0.196	0.008	0.181	0.213

AcdP, academic performance; SelE, self-efficacy; SocE, social and emotional skills; TeaS, teacher-student relationship.

**Table 6:** Mediational effect of indirect model.

Paths	Standardized coefficient	Bootstrap SE	Proportion of total effect
Indirect effect	0.059***	0.008	66.66%
Direct effect	0.030***	0.005	33.34%
Total effect	0.089***	0.010	100.00%

***p < 0.001.

## 5. Discussion

The associations between students’ academic success and their socioemotional skills have been understudied, and little is understood about the underlying mechanisms. The current study sought to test a theoretically derived structural equation model with a sample of students from low socioeconomic areas of China. The combination of the selected independent and mediational variables offered a powerful explanation for the variance in students’ social emotional outcomes in this sample. Overall, these findings extend the evidence of skill cross-productivity, whereby one type of skill can help foster other skills over time (Cunha et al., 2010). This study documents that cognitive skill is a salient predictor of non-cognitive skill in schoolchildren. Students who experienced higher academic achievement are more prone to positive socioemotional developmental outcomes is consonant with the findings of other investigations (OECD, 2015).

The findings also indicate that self-efficacy plays a significant role in mediating the positive relationship between academic achievement and socioemotional skills. This finding is consistent with social cognitive theory, which indicates that when individuals receive affirmative feedback, they may feel more competent (Bandura, 1986). Furthermore, students with high self-efficacy tend to feel motivated to engage in a task, and exhibit positive emotions in their school life (Bandura, 1997). Academic performance may be the primary source of positive feedback in schools for students from less-developed areas of China. Consequently, academic performance becomes a prominent and positive predictor of self-efficacy, which sequentially predicts socioemotional skills.

In addition, the analysis reveals that the positive effect of academic achievement on socioemotional skills is partially mediated by teacher-student relationships. This is in line with prior studies, which concluded that students who demonstrated stronger motivation and engagement received more involvement and support from their teachers (Skinner and Belmont, 1993), that the student’s academic performance influenced teacher’s perceptions of their students (Aluja-Fabregat et al., 1999) and that the teacher preferred students with higher performance (Wentzel and Asher, 1995; Kuklinski and Weinstein, 2000; Davis, 2006). This may have different implications when viewed through the lens of Chinese culture. Confucianism permeates many aspects of Chinese society, including to “produce” academically excellent students. These concepts are rooted in a cultural heritage, Chinese parents and school teachers value students’ academic performance; as a result, students who excel academically always maintain a good relationship with their parents and teachers (Dhingra and Manhas, 2009). Simultaneously, children’s interactions with their teachers can influence children’s social and emotional outcomes. The results were congruent with some previous studies in different scenarios. Harter (1999) found that children’s connections with their teachers can influence children’s self-concept, and that when teachers are affectionate, emotionally available, involved and supportive, children will acquire positive self-evaluations. Therefore, when dealing with students, teachers should instill greater self-consciousness of equity, care, non-discrimination.

Furthermore, the whole pattern of the results extends the existing interpretation of the interaction among family background, school environment, and students’ skills in general. Family environment and parents play a prominent role in shaping children’s skills is a consensus (Heckman and Kautz, 2013). School’s and teacher’s efforts might prevent children’s skills from being hampered by disadvantaged family backgrounds. Over the past half-century, western notions of education along with novel socialization goals have taken root inside urban and middle-class Chinese families, including respect for student’s curiosities and desires, highlighting individual differences, and fostering self-autonomy, while these notions do not appear to be widespread in rural and lower-class families (Kong, 2015).

## 6. Conclusion

To conclude, this study adds to a growing body of research concerning the linkages between academic performance and social and emotional skills, as well as the development of children from disadvantaged families.

First, a positive relationship between academic achievement and socioemotional skills was found. Increased academic performance was associated with higher self-efficacy, a better teacher-student relationship, and positive socioemotional developmental outcomes. Second, self-efficacy and teacher-student relationship partially mediated the association between academic achievement and socioemotional skills of elementary students, which will add to the literature as eastern evidence of the relationships of the existing theories. The overall pattern of the results suggests that schoolteachers play vital roles in restraining the inherent but negative influence of adverse family environments on children’s development. Hence, it appears important to encourage schools and teachers to provide students, particularly students with poor academic performance, with more comprehensive support in order to enrich social and emotional competence in primary school students in underdeveloped areas of China to bridge the gap in children’s outcomes between urban and rural areas. Because students with poor academic performance tend to have a lower level of self-efficacy and poorer relationships with teachers, they have inferior social–emotional competences. From a long-term perspective, institutions and policymakers should pay attention to both cognitive and non-cognitive skills in students with poor performance or low self-efficacy, which might buffer the loss of human capital.

Our results indicate two clear channels between academic performance and social–emotional skills. The first is through an intrapersonal characteristic--self-efficacy, the second is through an interpersonal factor--teacher-student relationship. This has implications for the design of future policies in disadvantaged regions. Improvement of students’ learning contexts means not just improving students’ performance, but also building favorable environments and teacher-student relationships, which are conducive to children’s social and emotional development for its long-lasting impact on life outcomes. Professional development and training programs can provides teachers with the necessary knowledge and skills to improve their communication, social and emotional skills, and better understand how to build positive relationships with their students (Darling-Hammond et al., 2017).

In China, for example, the governments and NGOs have implemented several teacher training programs to enhance teachers’ social–emotional learning skills. However, it is important to note that there are significant differences in educational resources across different regions, and one important aspect is the ability and effectiveness of teachers (Yang et al., 2014). And in rural areas of China, cultural expectations prioritize academic performance, which may hinder teachers’ attention to non-cognitive development of students. Therefore, training programs should be targeted at improving rural teachers’ awareness of the importance of students’ social and emotional development and enhancing their professional skills in guiding students’ social and emotional learning. This requires support from the education department, especially local education departments, to provide more assistance. At the same time, cooperation between schools and NGOs should be strengthened to provide more customized and personalized social and emotional skill learning support for schools, teachers and students.

## Data availability statement

The raw data supporting the conclusions of this article will be made available by the authors, without undue reservation.

## Ethics statement

The studies involving human participants were reviewed and approved by the Beijing Normal University. Written informed consent to participate in this study was provided by the participants’ legal guardian/next of kin.

## Author contributions

CH: formal analysis, data curation, writing—original draft, and writing—review and editing. XZ: conceptualization, methodology, investigation, writing—review and editing, and funding acquisition. All authors contributed to the article and approved the submitted version.

## Funding

This study was supported by the Beijing Cihong Charity Foundation (SKHX2019491) to XZ.

## Conflict of interest

The authors declare that the research was conducted in the absence of any commercial or financial relationships that could be construed as a potential conflict of interest.

## Publisher’s note

All claims expressed in this article are solely those of the authors and do not necessarily represent those of their affiliated organizations, or those of the publisher, the editors and the reviewers. Any product that may be evaluated in this article, or claim that may be made by its manufacturer, is not guaranteed or endorsed by the publisher.
